# Hearing loss in Korean adolescents: The prevalence thereof and its association with leisure noise exposure

**DOI:** 10.1371/journal.pone.0209254

**Published:** 2019-01-04

**Authors:** Jihye Rhee, Dongwook Lee, Hyun Jung Lim, Moo Kyun Park, Myung Whan Suh, Jun Ho Lee, Yun-Chul Hong, Seung-Ha Oh

**Affiliations:** 1 Departments of Otorhinolaryngology, Veterans Health Service Medical Center, Seoul, Korea; 2 Department of Preventive Medicine, Seoul National University College of Medicine, Seoul, Korea; 3 Department of Otorhinolaryngology-Head and Neck Surgery, Seoul National University Hospital, Seoul National University College of Medicine, Seoul, Korea; University of California Irvine, UNITED STATES

## Abstract

The increasing use of personal listening devices (PLDs) has been accompanied by a rise in the prevalence of hearing loss (HL) in younger age groups. However, there have been few reports on the relationship between HL and leisure noise exposure (LNE) in adolescents. The purpose of our study was to investigate the prevalence of HL in students attending general middle and high schools and to identify factors affecting HL prevalence. A total of 2,879 nationally representative adolescents in the first years of middle and high school underwent audiometric testing and otological examinations, and completed questionnaires, from June to December 2016. A speech-frequency hearing loss (SFHL) was considered present when the pure tone averages (PTAs) at 0.5, 1, and 2 kHz were ≥ 15 dB and a high-frequency hearing loss (HFHL) was considered present when the PTAs at 3, 4, 6, and 8 kHz were ≥ 15 dB. About 17% of Korean adolescents exhibited at least slight HL. The prevalence rates of SFHL and HFHL in the poorer ear were 11.6% and 10.3%, respectively, among Korean adolescents. The use of local area network (LAN) gaming centers and an experience of being asked by others to lower earphone volume were associated with both SFHL and HFHL. It is important to avoid excessive LNE to prevent adolescent HL. Additionally, SFHL or HFHL in the poorer ear was associated with lower academic performance.

## Introduction

Noise-induced hearing loss is a hearing impairment caused by exposure to loud sounds, and initially affects the high-frequency hearing threshold. Previously, noise-induced hearing loss that was attributable to occupational noise exposure was of major concern, and hearing loss (HL) caused by leisure noise exposure (LNE) was considered to be relatively unimportant [1, 2]. Although occupational HL is high in developing countries, the prevalence thereof is lower in developed countries because of governmental regulations, the use of personal protective equipment, and early HL detection via periodic screening [1, 3]. However, LNE is steadily increasing, attributable to major rises in smartphone use and participation in noisy leisure activities, such as listening to music through personal music systems and attending rock concerts or public nightclubs [3–6]. Noise exposure from repetitive leisure activities, which many young people are willing to accept, can increase the auditory threshold, particularly in this population [7–10].

A study from the USA reported that the prevalence of HL among US adolescents increased from 14.9% in 1988–1994 to 19.5% in 2005–2006. Although no significant relationship between noise exposure and HL was found, an influence of noise may be inferred by the reported increase in high-frequency hearing loss (HFHL) [11]. Another study did not find an increase in HL among US adolescents during these periods, but found an increase in the prevalence of noise-induced threshold shifts in female adolescents. Female participants used less hearing protection devices than males and the authors inferred that this may have some association with the increased prevalence of noise-induced threshold shifts [12]. Hong et al. also reported unilateral and bilateral HFHLs > 20 dB in 5.0% and 1.9%, respectively, of 1,658 adolescents aged 13–18 years in South Korea. They categorized the sources of noise as occupational, non- occupational, and momentary, and aimed to investigate how they affected HL. Although they did not detect a direct association between HL and any particular sort of noise, the use of earphones in noisy places was significantly associated with bilateral HFHL [13].

Thus, it has not yet been determined whether noise exposure affects adolescent HL. LNE is difficult to quantify and HL attributable to LNE varies from a temporary threshold shift to permanent HL. However, if noise exposure affects adolescent HL, early detection of HL and avoidance of additional noise exposure may be the most important factors to prevent further exacerbation.

In this study, we investigated the prevalence of HL and the associations between HL and noise-related factors, including specific types of LNE that adolescents are typically exposed to, as well as self-rated academic achievement in adolescents in South Korea.

## Materials and methods

### Study design and subjects

We performed a cross-sectional, complex sampling survey of first-year middle and high school students, except those attending special schools for disabled children. The sampling frame was the 2015 yearbook of educational statistics prepared by the Korean Educational Statistics Services [14].

We sought to enroll a total of 3,100 students from 124 middle schools and 124 high schools, based on the prevalence of HL in Korean adolescents, the estimated association between noise and HL, and an expected 80% response rate [15, 16]. The sample size required to analyze the association between LNE and HL was calculated using G*Power software, considering the sample size in a previous study, a statistical power of 0.80, and a two-tailed α-value of 0.05 [17]. We obtained a list of all Korean middle schools (n = 3,204), and another of all high schools (n = 2,383), and classified the schools for random sampling to ensure proportional representation of all regions of Korea (eight metropolitan and eight suburban or rural regions) and all types of schools (single-gender, coeducational, general, and vocational). Sample schools were systematically selected in each region from the lists of middle and high schools, in alphabetical order, followed by random selection of sample classes after ensuring that all classes were educationally similar. We targeted 248 of the 5,587 middle and high schools in Korea, expecting a 50% school response rate based on a pilot study, and that each class would have 25 students. Finally, sampling weight was calculated as the inverse selection probability for each stratum and each school. The final sampling weight was obtained after adjustment for non-response.

The survey was performed from June to November 2016. After distributing written information to students and parents, we evaluated only those who provided written informed consent of themselves and their parents. This study was approved by the Institutional Review Board of Seoul National University Hospital (No. 1604-086-755).

### Data collection and measurements

#### Questionnaire

Students and parents were asked to complete separate questionnaires. Both questionnaires were based on Forms 1–2 and 1–3 of the Enforcement Rule for Health Screening at School of the School Health Act of South Korea, and those of the Korea Nutrition and Health Examination Survey (a nationally representative South Korean survey assessing the health and nutritional status of the entire population [18]).

The student questionnaire explored gender, age, any history of a disorder of the ear, nose, or throat, subjective hearing status, and any tinnitus. Participants answered “yes”, “no”, or “I don’t know” to the following question about tinnitus: “Have you ever experienced tinnitus, such as beeping, ringing, or buzzing, in the past year?” Questions pertaining to personal listening devices (PLDs) concerned usage of a PLD (“Do you use a personal listening device such as a smartphone, MP3 player, or personal media player?”), usage period (“How old were you when you started using a PLD?”), requests to turn down the volume (“Have you ever been asked to turn down the volume on your PLD?”), and the subjective volume intensity (10-point Likert scale), based on a previous study by Cone et al. [15]. In terms of LNE, we asked how often the subject visited local area network (LAN) gaming centers, karaoke rooms, and concert halls. We explored drinking and smoking histories. We asked the subject to self-rate his/her academic performance to allow us to explore the association between school performance and hearing. Academic performance was reported using a five-point Likert scale (Excellent, Good, Normal, Bad, or Poor). Participants who responded “Normal”, “Bad”, or “Poor” were considered to exhibit lower academic performance. The parental questionnaires explored the student’s perinatal history, any visit to an intensive care unit, any family history of HL, and the residential noise level.

#### Audiological evaluation

Pure tone audiometry was conducted using an AD229b diagnostic audiometer (Interacoustics, Assen, Denmark) in soundproof booths inside a mobile vehicle by four experienced audiologists. Prior to audiometry, otological examinations were performed by trained, experienced otolaryngology residents using otoscopes. Factors affecting hearing, such as external ear anomalies, ear wax, retraction of the tympanic membrane, cholesteatoma, and middle ear effusion were recorded; modifiable factors (cerumen or foreign bodies in the external auditory canal) were managed prior to the hearing tests.

The tested frequencies ranged from 0.5 to 8 kHz, i.e., 0.5, 1, 2, 3, 4, 6, and 8 kHz. Testing began at 1 kHz, followed by 2, 4, 8, 1, 0.5, and 0.25 kHz. The participants started the test by listening to a 30 dB tone; the test tone was reduced by 10 dB or increased by 5 dB according to the presence or absence of a response. The hearing threshold was defined as the lowest intensity level (in dB) at which the participant responded to 50% of the stimuli. When the hearing threshold was ≥ 25 dB at any frequency (0.5–8 kHz), the bone conduction threshold to distinguish between sensorineural and conductive HL was determined. The severity of HL was based on the poorer threshold for unilateral HL and the better threshold for bilateral HL, as described previously [11]. We defined “slight HL” as a hearing threshold ≥ 15 dB and “worse HL” as a hearing threshold ≥ 25 dB, thus, including mild HL. The speech-frequency hearing loss (SFHL) was the average of the HLs at 0.5, 1, and 2 kHz, while the HFHL was the average of the HLs at 3, 4, 6, and 8 kHz.

### Statistical analyses

First, we calculated the unweighted frequencies and weighted prevalence rates of SFHL and HFHL; this was deemed appropriate given the multi-stage, complex sampling design. Second, we used a multivariate logistic regression model to calculate odds ratios (ORs) for the associations between HL and various factors. The model included demographic characteristics (educational level, gender, and monthly household income); known risk factors (cigarette smoking, alcohol consumption, a history of sinusitis, the use of sinusitis medication, a history of otitis media, and the use of otitis media medication); tinnitus status (“yes”, “no”, or “I don’t know”); and associations between HL and noise sources (PLD use, duration of such use, PLD volume, requests from others to turn down the volume, exposure to leisure noise over the past year). To estimate the association between leisure time noise exposure and HL with consideration of known risk factors, noise sources were not included as model covariates. We adjusted for potential confounders (known risk factors for HL) including age, gender, household income, smoking status, alcohol consumption, any history of ear infection or rhinosinusitis, and any family history of HL [11]. Third, we analyzed the dose-response associations between HL and use of LAN gaming centers, a major source of LNE in Korea. We multiplied visits per month by hours per visit by years since first visit; this was the cumulative LNE imparted by the gaming centers. We divided all subjects into three groups in terms of LAN gaming center use: never-user, light user (lower 50%), and heavy user (upper 50%). We employed multivariate logistic regression to explore the association between LAN gaming center exposure and HL, adjusted for age, gender, household income, smoking status, alcohol use, any history of ear infection or rhinosinusitis, and any family history of HL. Finally, we performed a multivariate logistic regression to investigate the associations between lower academic performance and hearing status after adjusting for education level, gender, and household income. All statistical analyses were performed with the aid of SAS SURVEYMEANS, SAS SURVEYFREQ, or SAS SURVEYLOGISTIC (ver. 9.3; SAS Institute, Cary, NC) to allow for the complex survey design and to consider the sampling weights. Statistical significance was defined as a two-sided *p-*value < 0.05.

## Results

**Fig 1:**
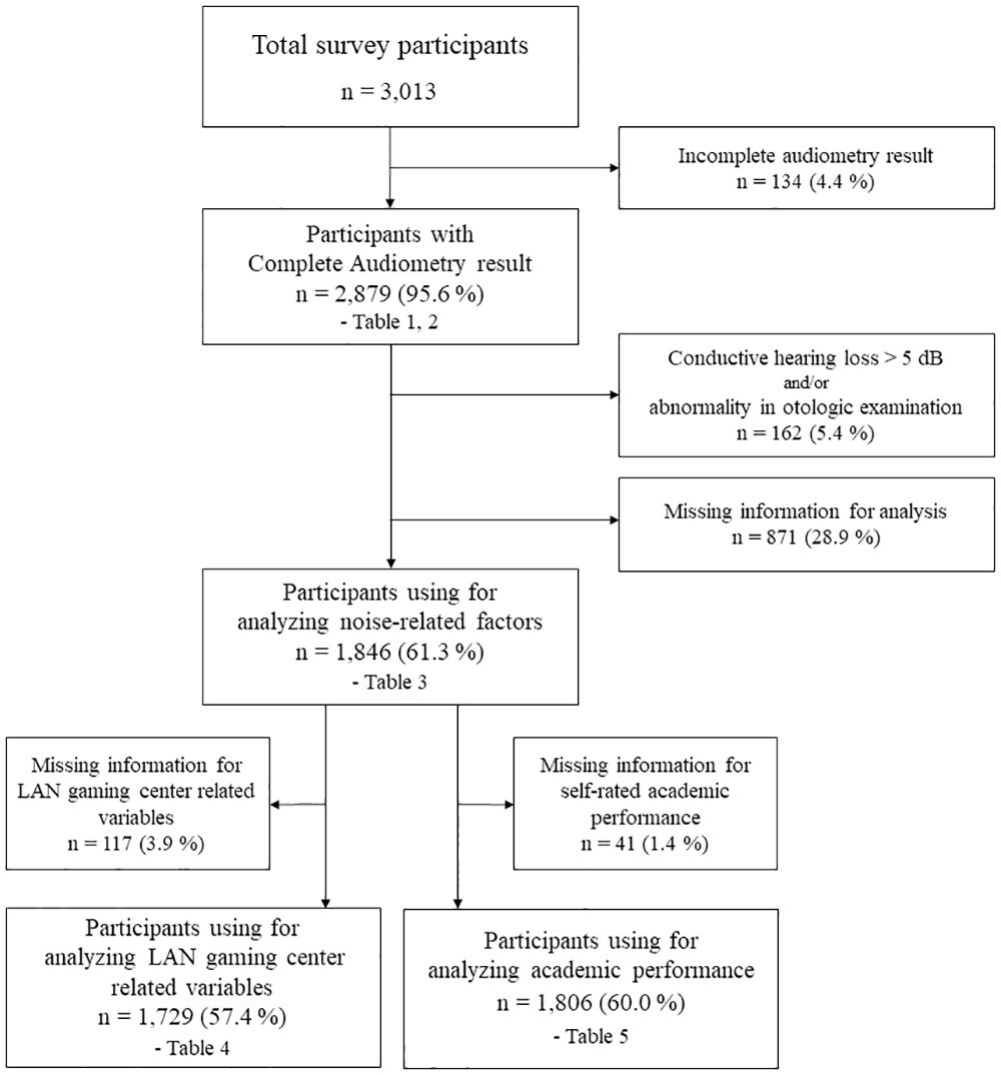
Flow chart of the selection process.

Overall, 109 of the 248 schools contacted agreed to participate in the survey, with 3,013 students agreeing to complete the questionnaires. Of the 3,013 participants, 134 were excluded because of incomplete data. A total of 2,879 students underwent audiological and otological examinations (95.6%). The demographic characteristics of these 2,879 participants are summarized in Table 1. We excluded those with conductive HL > 5 dB and/or any otological abnormality (middle ear effusion, cholesteatoma, and/or excessive cerumen). Age, gender, smoking habits, alcohol intake, socioeconomic status, and noise-related behaviors did not differ significantly between participants who were included and excluded. After excluding those for whom essential data were missing, 1,846 students were assessed for factors related to HL; 1,803 students were included to analyze factors related to tinnitus; 1,729 students were subjected to analysis regarding LAN gaming center-related variables; and 1,806 students were subjected to an academic performance analysis (Fig 1).

**Table 1:** Demographic characteristics of study participants by school level.

	Total^§^	Middle school, Grade 7		High school, Grade 7	
		Male^§^	Female^§^	Male^§^	Female^§^
**Total**	2,879 (100.0)	852 (100.0)	720 (100.0)	659 (100.0)	648 (100.0)
**Household income per month, $US**					
< 2,000	77 (2.3)	22 (2.7)	17 (2.8)	22 (1.4)	16 (2.1)
2,000–3,000	323 (9.8)	77 (7.4)	79 (10.1)	80 (10.1)	87 (12.2)
3,000–4,000	1,133 (39.2)	338 (35.9)	293 (40.9)	236 (39.9)	266 (40.6)
≥ 4,000	962 (35.9)	309 (38.9)	259 (36.9)	195 (33.7)	199 (33.8)
Missing	384 (12.8)	106 (15.1)	72 (9.4)	126 (15.0)	80 (11.3)
**Cigarette smoking status**					
Never	2,534 (89.0)	754 (91.5)	677 (95.1)	493 (76.0)	610 (94.1)
Ever	323 (10.8)	82 (7.9)	37 (4.6)	166 (24.0)	38 (5.9)
Missing	22 (0.2)	16 (0.6)	6 (0.2)	0 (0)	0 (0)
**Alcohol use**					
Never	2,310 (81.0)	756 (89.3)	665 (93.6)	420 (64.6)	469 (75.4)
Ever	543 (18.8)	77 (10.0)	48 (6.1)	239 (35.4)	179 (24.6)
Missing	26 (0.3)	19 (0.7)	7 (0.3)	0 (0)	0 (0)
**History of sinusitis**					
Yes	265 (9.3)	63 (9.1)	49 (5.9)	85 (12.2)	68 (10.3)
No	2,358 (81.5)	687 (78.5)	616 (87.8)	518 (78.5)	537 (81.2)
Missing	256 (9.2)	102 (12.4)	55 (6.4)	56 (9.3)	43 (8.4)
**Took medication for sinusitis in past 2 weeks**					
Yes	41 (1.5)	13 (2.0)	9 (0.9)	11 (2.4)	8 (0.8)
No	2,299 (80.2)	667 (78.0)	593 (84.9)	525 (80.7)	514 (76.7)
Missing	539 (18.3)	172 (20.0)	118 (14.2)	123 (16.9)	126 (22.5)
**History of ear infection**					
Yes	295 (9.8)	69 (7.5)	78 (10.3)	67 (8.8)	81 (13.4)
No	2,317 (80.4)	677 (79.0)	590 (83.9)	535 (81.5)	515 (76.7)
Missing	267 (9.8)	106 (13.5)	52 (5.8)	57 (9.7)	52 (9.9)
**Took medication for otitis media in past 2 weeks**					
Yes	21 (0.6)	9 (0.7)	7 (1.0)	2 (0.1)	3 (0.5)
No	2,307 (80.4)	667 (77.6)	597 (85.1)	531 (82.7)	512 (75.8)
Missing	551 (19.0)	176 (21.7)	116 (13.9)	126 (17.1)	133 (23.8)
**Subjective hearing discomfort**					
None	2,279 (79.5)	689 (81.3)	568 (80.8)	522 (77.9)	500 (77.7)
Mild-to-moderate	427 (15.2)	94 (11.8)	105 (13.8)	108 (18.0)	120 (17.5)
Severe	11 (0.4)	1 (0.1)	1 (0.1)	5 (0.6)	4 (0.7)
Profound					
Missing	162 (4.9)	68 (6.7)	46 (5.2)	24 (3.4)	24 (4.1)
**Has hearing aid**					
Yes	14 (0.2)	9 (0.6)	3 (0.2)	1 (0.02)	1 (0.1)
Yes, but do not use	4 (0.02)	1 (0.04)	1 (0.04)	1 (0.002)	1 (0)
No	885 (30.3)	233 (26.1)	196 (25.3)	230 (36.8)	226 (33.8)
Missing	1,976 (69.4)	609 (73.3)	520 (74.5)	427 (63.2)	420 (66)
**Tinnitus**					
Yes	1,298 (43.9)	247 (27.0)	319 (45.0)	310 (45.0)	422 (62.6)
No	1,035 (37.5)	430 (53.8)	240 (33.9)	232 (37.3)	133 (21.5)
I don’t know	416 (14.6)	116 (13.8)	126 (17.7)	90 (13.1)	84 (13.8)
Missing	130 (4.0)	59 (5.4)	35 (3.3)	27 (4.7)	9 (2.2)
**Personal stereo usage**					
Yes	2,518 (87.1)	666 (76.8)	638 (89.2)	596 (89.7)	618 (94.3)
No	345 (12.8)	175 (22.8)	77 (10.6)	63 (10.3)	30 (5.7)
Missing	16 (0.2)	11 (0.4)	5 (0.2)	0 (0)	0 (0)
**Type of personal stereo**					
With earphones	2,330 (80.8)	575 (67.6)	596 (83.0)	553 (83.0)	606 (92.3)
Any other device With headphones	152 (5.2)	77 (8.0)	30 (4.7)	36 (5.8)	9 (1.8)
Not applicable	361 (12.9)	186 (23.2)	82 (10.8)	63 (10.3)	30 (5.7)
Missing	36 (1.0)	14 (1.2)	12 (1.5)	7 (1.0)	3 (0.2)
**Usage period**					
Not applicable	345 (12.8)	175 (22.8)	77 (10.6)	63 (10.3)	30 (5.7)
0–4 years	1,379 (48.3)	499 (57.7)	421 (58.7)	269 (41.9)	190 (31.8)
≥ 4 years	1,139 (38.8)	167 (19.1)	217 (30.5)	327 (47.8)	428 (62.5)
Missing	16 (0.2)	11 (0.4)	5 (0.2)	0 (0)	0 (0)
**Volume level**					
Not applicable	345 (12.8)	175 (22.8)	77 (10.6)	63 (10.3)	30 (5.7)
0–4	1,446 (52.2)	375 (42.3)	384 (56.3)	336 (55.9)	351 (55.5)
5–9	1,072 (34.8)	291 (34.5)	254 (32.9)	260 (33.7)	267 (38.8)
Missing	16 (0.2)	11 (0.4)	5 (0.2)	0 (0)	0 (0)
**Request to turn down the volume**					
Ever experienced	401 (14.0)	76 (9.4)	121 (18.3)	88 (12.5)	116 (16.8)
Never experienced	2,062 (71.4)	572 (65.7)	497 (69.2)	500 (75.9)	493 (75.7)
Missing	416 (14.6)	204 (24.9)	102 (12.6)	71 (11.6)	39 (7.5)
**Usage of noisy facility in the past year**				
LAN gaming center	1,701 (59.8)	568 (66.1)	224 (31.8)	576 (88.6)	333 (50.4)
Karaoke room	2072 (74.7)	392 (48.5)	624 (87.7)	482 (76.9)	574 (89.7)
Concert auditorium	370 (12.3)	50 (6.2)	114 (14.8)	60 (7.3)	146 (23.2)
Dance club	8 (0.3)	1 (0.02)	1 (0.04)	4 (1.0)	2 (0.3)

^§^ Unweighted frequencies with weighted percentages.

The prevalence rates of SFHL and HFHL were 11.6% and 10.3%, respectively, in Korean adolescents. In total, 17.2% exhibited either SFHL or HFHL. The prevalence of any HL (PTA of either ear better at high frequencies [3, 4, 6, and 8 kHz] or speech frequencies [0.5, 1, and 2 kHz] ≥ 15 dB) were 17.9% and 16.5% among middle and high school students, respectively. The prevalence rates of any HL at high frequencies were 10.5% among middle school students and 10.2% among high school students. The prevalence rates of any HL at speech frequencies were 12.7% and 10.4% among middle and high school students, respectively. The prevalence of slight HL was 16.0% and that of serious HL was 1.2%. The detailed data are shown in Table 2.

**Table 2:** Prevalence of hearing loss in Korean adolescents.

	Slight or serious (≥ 15 dB)		Slight (15–24 dB)		Serious (≥ 25 dB)	
	No.	Prevalence, % (95% CI)	No.	Prevalence, % (95% CI)	No.	Prevalence, % (95% CI)
All participants (n = 2,879)						
Any* HL^§^	570	17.2 (15.0–19.4)	527	16.0 (14–18)	43	1.2 (0.8–1.6)
Any HFHL	330	10.3 (8.8–11.9)	295	9.4 (7.9–10.9)	35	1.0 (0.7–1.2)
Any SFHL	391	11.6 (9.5–13.6)	369	10.9 (9.0–12.8)	22	0.7 (0.3–1.0)
Bilateral HL^§^	181	6.6 (5.4–7.7)	164	5.9 (4.7–7.1)	17	0.7 (0.5–0.9)
Bilateral HFHL	102	3.6 (2.8–4.4)	85	2.9 (2.1–3.7)	17	0.7 (0.5–0.9)
Bilateral SFHL	118	4.3 (3.1–5.5)	111	4.0 (2.8–5.2)	7	0.3 (0.1–0.5)
Middle school, Grade 7 (n = 1,572)						
Any* HL^§^	329	17.9 (14.4–21.4)	307	16.7 (13.5–19.9)	22	1.2 (0.6–1.7)
Any HFHL	183	10.5 (8.6–12.4)	168	9.7 (7.8–11.6)	15	0.8 (0.5–1.2)
Any SFHL	243	12.7 (9.5–15.8)	226	11.7 (8.8–14.5)	17	1.0 (0.4–1.5)
Bilateral HL^§^	108	6.5 (5.3–7.7)	101	6.0 (4.8–7.3)	7	0.4 (0.2–0.7)
Bilateral HFHL	57	3.8 (3.1–4.5)	50	3.3 (2.6–4.1)	7	0.4 (0.2–0.7)
Bilateral SFHL	76	4.4 (3.2–5.6)	72	4.0 (2.8–5.2)	4	0.3 (0.1–0.6)
High school, Grade 10 (n = 1,307)						
Any* HL^§^	241	16.5 (13.8–19.1)		15.2 (12.7–17.7)	21	1.3 (0.7–1.9)
Any HFHL	147	10.2 (7.7–12.7)	127	9.0 (6.5–11.6)	20	1.2 (0.7–1.6)
Any SFHL	148	10.4 (7.6–13.1)	143	10.0 (7.5–12.5)	5	0.4 (0–0.8)
Bilateral HL^§^	73	6.7 (4.5–8.8)	63	5.8 (3.5–8.0)	10	0.9 (0.5–1.3)
Bilateral HFHL	45	3.4 (1.8–4.9)	35	2.5 (0.9–4.0)	10	0.9 (0.5–1.3)
Bilateral SFHL	42	4.2 (2.0–6.5)	39	4.0 (1.8–6.2)	3	0.2 (0–0.6)

* The PTA in the most affected ear was used to define any HL, and the PTA in the less affected ear used to define bilateral HL. Any HL was considered present if bilateral HL was evident at either high or speech frequencies.

^§^ Participants exhibiting HL either at high frequencies (3, 4, 6, and 8 kHz) or speech frequencies (0.5, 1, and 2 kHz) were classified into these categories.

HL: hearing loss; CI: confidence interval; PTA: pure tone average; HFHL: high-frequency hearing loss; SFHL: speech-frequency hearing loss.

We constructed multivariate logistic regression models to analyze the associations between HL and related factors (Table 3). All analyses were performed after adjusting for education level, gender, household income, cigarette smoking status, alcohol consumption, a history of sinusitis or otitis media (n = 1,846). Education level was not associated with bilateral HL, bilateral HFHL, or bilateral SFHL. Gender was not associated with any HL or HFHL, but female gender was negatively associated with SFHL (OR, 0.49; 95% CI, 0.29–0.82). Subjects who had ever drunk alcohol were at risk of HFHL (OR, 1.90; 95% CI, 1.10–3.28). A history of rhinosinusitis was associated with SFHL and a history of ear infection was associated with HFHL. A low household income was associated with HL. Those living in households with incomes of < $2,000 per month were 8.5-fold more likely to exhibit HFHL (OR, 8.50; 95% CI, 4.84–14.93). We found no significant difference in the prevalence of HL based on the extent or duration of PLD usage. However, among respondents who had ever been asked to lower their earphone volume, the risk of HFHL was 3.32-fold higher (95% CI, 2.09–5.25). Subjective volume was not associated with HL. Recent use of LAN gaming centers increased HFHL 2.21-fold (95% CI, 1.15–4.23). Those who attended concerts exhibited a lower prevalence of HFHL than those who did not attend concerts (OR, 0.35; 95% CI, 0.16–0.77). Visiting karaoke rooms was not associated with HFHL prevalence.

**Table 3:** Multivariate logistic regression of potential risk factors for HL in Korean adolescents (n = 1,846).

	No.	Bilateral HL*		Bilateral HFHL*		Bilateral SFHL*	
		n (%) ^§^	OR (95% CI) ^†^	n (%) ^§^	OR (95% CI)	n (%) ^§^	OR (95% CI)
**School level**							
Middle school, Grade 7	1,034	66 (5.7)	1 (Reference)	31 (3.0)	1 (Reference)	46 (3.9)	1 (Reference)
High school, Grade 10	812	42 (5.0)	0.83 (0.58–1.20)	23 (2.4)	0.74 (0.41–1.33)	26 (3.5)	0.80 (0.53–1.22)
**Gender**							
Male	937	59 (6.2)	1 (Reference)	23 (2.8)	1 (Reference)	45 (4.7)	1 (Reference)
Female	909	49 (4.5)	0.63 (0.42–0.95)	31 (2.6)	0.84 (0.51–1.40)	27 (2.7)	**0.49 (0.29–0.82)**
**Household income per month, $US**							
< 2,000	47	5 (15.7)	**5.17 (3.29–8.11)**	3 (14.3)	**8.50 (4.84–14.93)**	3 (12.3)	**7.98 (4.71–13.54)**
2,000–3,000	241	12 (3.6)	1.05 (0.55–2.01)	5 (1.3)	0.58 (0.30–1.10)	10 (3.3)	**2.22 (1.05–4.68)**
3,000–4,000	840	58 (6.9)	**2.03 (1.24–3.33)**	27 (2.9)	1.29 (0.68–2.43)	41 (5.1)	**3.39 (1.84–6.23)**
≥ 4,000	718	33 (3.6)	1 (Reference)	19 (2.2)	1 (Reference)	18 (1.7)	1 (Reference)
**Cigarette smoking**							
Never	1,689	97 (5.5)	1 (Reference)	49 (2.8)	1 (Reference)	64 (3.7)	1 (Reference)
Ever	157	11 (4.3)	0.74 (0.41–1.33)	5 (1.8)	0.58 (0.27–1.28)	8 (3.3)	0.79 (0.41–1.54)
**Alcohol consumption**							
Never	1,552	88 (5.4)	1 (Reference)	40 (2.5)	1 (Reference)	60 (3.8)	1 (Reference)
Ever	324	20 (5.2)	0.98 (0.65–1.48)	14 (3.8)	**1.90 (1.10–3.28)**	12 (3.4)	0.82 (0.56–1.20)
**History of sinusitis**							
Yes	173	11 (6.3)	0.99 (0.68–1.46)	5 (4)	0.98 (0.62–1.56)	10 (6.2)	**1.68 (1.16–2.44)**
No	1,673	97 (5.3)	1 (Reference)	49 (2.6)	1 (Reference)	62 (3.4)	1 (Reference)
**Took medication for sinusitis in the past 2 weeks**							
Yes	19	1 (4.1)	0.77 (0.47–1.26)	1 (4.1)	**2.10 (1.09–4.03)**	1 (4.1)	0.66 (0.39–1.11)
No	1,827	107 (5.4)	1 (Reference)	53 (2.7)	1 (Reference)	71 (3.7)	1 (Reference)
**History of ear infection**							
Yes	195	13 (8.2)	**1.92 (1.14–3.22)**	7 (5.7)	**2.98 (1.53–5.81)**	9 (5.7)	1.73 (0.99–3.02)
No	1,651	95 (5.1)	1 (Reference)	47 (2.4)	1 (Reference)	63 (3.5)	1 (Reference)
**Took medication for otitis media in the past 2 weeks**							
Yes	8	0 (0)		0 (0)		0 (0)	
No	1838	108 (5.4)	1 (Reference)	54 (2.7)	1 (Reference)	72 (3.7)	1 (Reference)
**Tinnitus**^**#**^							
Yes	968	51 (5.4)	1 (Reference)	24 (2.8)	1 (Reference)	33 (3.4)	1 (Reference)
No	835	53 (5.4)	1.01 (0.74–1.39)	29 (2.7)	0.86 (0.54–1.36)	36 (4.0)	1.24 (0.89–1.74)
**Personal stereo use**							
No	190	19 (8.0)	1 (Reference)	8 (3.6)	1 (Reference)	11 (4.4)	1 (Reference)
Yes	1,656	89 (5.1)	0.67 (0.35–1.26)	46 (2.6)	0.68 (0.20–2.38)	61 (3.6)	0.96 (0.49–1.91)
**Usage period**							
< 4 years	895	47 (4.8)	1 (Reference)	23 (2.7)	1 (Reference)	34 (3.6)	1 (Reference)
≥ 4 years	761	42 (5.4)	1.28 (0.84–1.94)	23 (2.4)	0.94 (0.53–1.66)	27 (3.6)	1.16 (0.79–1.71)
Not applicable	190	19 (8)	1.65 (0.89–3.05)	8 (3.6)	1.43 (0.44–4.64)	11 (4.4)	1.10 (0.56–2.15)
**Volume level**							
0–4	941	42 (4.7)	1 (Reference)	20 (2.3)	1 (Reference)	30 (3.8)	1 (Reference)
5–9	715	47 (5.7)	1.34 (0.91–1.96)	26 (3.1)	1.46 (0.94–2.26)	31 (3.4)	0.99 (0.61–1.59)
Not applicable	190	19 (8.0)	1.70 (0.88–3.28)	8 (3.6)	1.72 (0.51–5.80)	11 (4.4)	1.04 (0.51–2.12)
**Request to turn down the volume**^††^							
Ever experienced	270	24 (6.7)	**1.67 (1.12–2.48)**	17 (5.3)	**3.32 (2.09–5.25)**	14 (3.2)	1.04 (0.58–1.84)
Never experienced	1,360	64 (4.8)	1 (Reference)	28 (2.0)	1 (Reference)	46 (3.7)	1 (Reference)
**Usage of LAN gaming center in the past year**							
Yes	1,084	68 (5.7)	0.95 (0.60–1.52)	37 (3.4)	**2.21 (1.15–4.23)**	45 (3.7)	0.67 (0.40–1.12)
No	762	40 (5)	1 (Reference)	17 (1.7)	1 (Reference)	27 (3.7)	1 (Reference)
**Use of karaoke room in the past year**							
Yes	1,364	81 (5.4)	1.35 (0.91–2.02)	41 (2.9)	1.59 (0.96–2.64)	53 (3.5)	1.11 (0.79–1.56)
No	482	27 (5.3)	1 (Reference)	13 (2.3)	1 (Reference)	19 (4.4)	1 (Reference)
**Attended a concert in the past year**							
Yes	257	12 (3.2)	0.65 (0.36–1.16)	5 (1.0)	**0.35 (0.16–0.77)**	9 (2.4)	0.85 (0.42–1.72)
No	1,589	96 (5.7)	1 (Reference)	49 (3.0)	1 (Reference)	63 (3.9)	1 (Reference)
**Attended a dance club in the past year**							
Yes	3						
No	1,843	108 (5.4)	1 (Reference)	54 (2.7)	1 (Reference)	72 (3.7)	1 (Reference)

* The PTA in the least-affected ear was used to categorize HL. HFHL was defined as PTAs at 0.5, 1, and 2 kHz ≥ 15 dB. SFHL was defined as PTAs at 3, 4, 6, and 8 kHz ≥ 15 dB. HL was considered present if bilateral HL was evident at either high or speech frequencies.

^§^ Unweighted frequencies with weighted percentages.

^†^ The ORs for HL were calculated using multivariate logistic regression after adjusting for school level (= age), gender, household income, cigarette smoking and alcohol consumption status, a history of sinusitis, a history of otitis media, and a history of HL in the family.

^††^ Missing data (n = 216) was not included in this analysis.

^**#**^ Missing data (n = 43) was no included in this analysis.

HL: hearing loss; HFHL: high-frequency hearing loss; SFHL: speech-frequency hearing loss; CI: confidence interval; PTA: pure tone average; OR: Odds Ratio.

Analysis of LAN gaming center use and HFHL prevalence showed a positive correlation between cumulative hours spent in gaming centers and HFHL prevalence (Table 4). Compared with never-users, the ORs for light users (bottom 50% of users) and heavy users (top 50%) were 1.57-fold (95% CI, 0.85–2.90) and 3.21-fold (95% CI, 1.60–6.88), respectively. The test for trends in cumulative use time was statistically significant (*p =* 0.003). However, no significant association was found between LAN use time and SFHL. The prevalence of HFHL was affected by the average use frequency (heavy users [7.7 ± 5.2 times]: OR, 2.43; 95% CI, 1.17–5.03) and time spent per visit (heavy users [2.6 ± 1.0 h]: OR, 2.14; 95% CI, 1.05–4.33).

**Table 4:** Multivariate logistic regression of the association between LAN gaming center-related variables and HL in Korean adolescents (n = 1,729).

Classification	n	Mean ± SD	HFHL*				SFHL*			
			n	%^§^	OR	95% CI	n	%^§^	OR	95% CI
**Years from first use**										
Never-user	762		17	1.7			27	3.7		
Lower 50%	526	1.9 ± 0.9	17	3.5	**2.01**	**(1.05–3.85)**	24	3.56	0.612	(0.34–1.12)
Higher 50%	441	5.8 ± 1.9	16	3.3	**2.39**	**(1.15–4.95)**	17	3.82	0.692	(0.41–1.18)
*p*-value					**0.014**				0.153	
**Number of visits per month - Usage number per month**										
Never-user	762		17	1.7			27	3.7		
Lower 50%	475	1.3 ± 0.6	14	3.1	1.95	(0.98–3.90)	14	3.2	0.67	(0.36–1.25)
Higher 50%	492	7.7 ± 5.2	19	3.7	**2.43**	**(1.17–5.03)**	27	4.2	0.61	(0.35–1.07)
*p-*value					**0.017**				0.090	
**Hours spent at any one visit - Usage hours at one time**										
Never-user	762		17	1.7			27	3.7		
Lower 50%	318	1.0 ± 0.2	4	3.4	**2.12**	**(1.14–3.92)**	7	3.9	0.72	(0.43–1.22)
Higher 50%	649	2.6 ± 1.0	29	3.4	**2.14**	**(1.05–4.33)**	34	3.6	0.60	(0.33–1.09)
*p-*value					**0.035**				0.095	
**Cumulative usage time**										
Never-user	762		17	1.7			27	3.7		
Light user (lower 50%)	475	62.8 ± 49.4	12	2.5	1.57	(0.85–2.90)	14	4.0	0.80	(0.45–1.41)
Heavy user (higher 50%)	492	982.2 ± 1,158.0	21	4.2	**3.21**	**(1.60–6.88)**	27	3.4	**0.49**	**(0.27–0.87)**
*p-*value					**0.003**				**0.019**	

* The PTA in the better ear was used to categorize HL. HFHL was defined as PTAs at 0.5, 1, and 2 kHz ≥ 15 dB. SFHL was defined as PTAs of 3, 4, 6, and 8 kHz ≥ 15 dB.

^§^ Unweighted frequencies with weighted percentages.

HL: hearing loss; HFHL: high-frequency hearing loss; SFHL: speech-frequency hearing loss; CI: confidence interval; PTA: pure tone average; OR: odds ratio.

HL affected school performance. In children with ≥ 15 dB of HFHL or SFHL, the odds of lower academic performance were 1.33-fold (95% CI, 1.02–1.74) and 1.47-fold (95% CI, 1.21–1.79), respectively, higher than those of other children (Table 5).

**Table 5:** Adjusted ORs for self-rated lower academic performance by the extent of HL. (n = 1,806).

Type of HL	No.	Self-rated poor academic performance	
		n (%) ^§^	OR (95% CI) ^†^
Any HL*			
Normal	1,483	798 (46.8)	1 (Reference)
Abnormal	323	199 (62.6)	**1.46 (1.12–1.77)**
Any HFHL*			
Normal	1,631	887 (54.0)	1 (Reference)
Abnormal	175	110 (61.1)	**1.33 (1.02–1.74)**
Any SFHL*			
Normal	1,579	856 (53.6)	1 (Reference)
Abnormal	227	141 (63.1)	**1.47 (1.21–1.79)**
Bilateral HL**			
Normal	1,701	925 (53.9)	1 (Reference)
Abnormal	105	72 (66.8)	**1.65 (1.10–2.47)**
Bilateral HFHL**			
Normal	1,753	959 (54.3)	1 (Reference)
Abnormal	53	38 (65.1)	1.60 (0.94–2.72)
**Bilateral SFHL**			
Normal	1,736	951 (54.2)	1 (Reference)
Abnormal	70	46 (66.0)	1.44 (0.95–2.17)

* The PTA in the poorer ear was used to categorize HL. HFHL was defined as PTAs at 0.5, 1, and 2 kHz ≥ 15 dB. SFHL was defined as PTAs at 3, 4, 6, and 8 kHz ≥ 15 dB.

** The PTA in the better ear was used to categorize HL. HFHL was defined as PTAs at 0.5, 1, and 2 kHz ≥ 15 dB. SFHL was defined as PTAs at 3, 4, 6, and 8 kHz ≥ 15 dB.

^§^ Unweighted frequencies with weighted percentages.

^†^ ORs for HL were calculated by multivariate logistic regression after adjusting for school level, gender, and household income.

HL: hearing loss; HFHL: high-frequency hearing loss; SFHL: speech-frequency hearing loss; CI: confidence interval; PTA: pure tone average; OR: odds ratio.

## Discussion

We found that the prevalence of HL was 17.2% overall, and 17.9% and 16.5% in middle and high schools, respectively. The extent of HFHL was 10.3%. LNE in noisy LAN gaming centers and setting earphone volume to levels causing others to ask that the volume be reduced affected HFHL. Both SFHL and HFHL were inversely associated with self-rated academic performance.

Currently, many adolescents use smartphones, which are the most common PLDs. In 2011, adolescent Korean smartphone users constituted 12.8% and 21.7% of all middle and high school students, respectively, but those figures rose to 86.6% and 90.2% in 2015 [19, 20]. The dramatic increase in accessibility to PLDs, and the resulting increase in LNE, can also be expected to affect the prevalence of noise-induced HL. We found no significant relationship between PLD use and HL. This seems to be due to variation in the interpretation of the question of whether or not a participant used a PLD. To determine whether the respondents used a PLD, the authors asked the following question.

“Do you use portable music and video playback devices, such as smart phones, MP3 players, or PMPs?”

Participants who had a smartphone for a purpose other than listening to music were also likely to answer “yes” and be misclassified as PLD users, which obscured the relationship between PLD use and HL. However, the following questions that concerned the precise pattern of PLD use showed that the requests to lower the volume seemed to be associated with greater HL. Therefore, the volume of LNE may be closely related to HL. This volume ranges from 50–100 dB. Use of PLDs at volumes > 80 dB LAeq increased hearing thresholds and affected syllable identification in a noisy environment [5]. We did not measure volume objectively. Rather, we recorded subjective volume intensities but found no relationship between self-reported volume and HL, which may indicate that self-reported volume data are inaccurate. However, we found a significant correlation between requests to lower the volume and HFHL, suggesting that the reactions of others to volume may be a simple and relatively accurate measure of volume control, and may be more reliable than self-reported volume.

We investigated the relationships between HL and several types of LNE: LAN gaming centers, karaoke rooms, and concert auditoria. The prevalence of HFHL was higher in adolescents using LAN gaming centers, in contrast to the findings of a recent report indicating that no significant relationship was evident between HL and the maximum equivalent LNE, such as time spent in nightclubs or music venues, at concerts or festivals, and listening through headphones [8]. Shin et al. explored noise exposure in Korean LAN gaming centers by installing noise dosimeters in the headsets of middle- and high-school students visiting such centers [21]. The average noise level was 80–85 dBA, and 6.7% of subjects were exposed to an average noise level >100 dBA, suggesting that the noise levels in gaming centers are high. Additionally, the LNEs of LAN gaming centers are repetitive and long. A total of 43.7% of our subjects reported that they visited such centers more than once a week; of these, >95% stated that they remained for >1 h on each visit. Frequent users (the top 50%) reported 7.7±5.2 visits/month and heavy users (the top 50%) remained for 2.6±1.0 h per visit. Of all students who used gaming centers, the number of visits per month, the length (in h) of any one visit, and cumulative visit time, were positively associated with HFHL. Therefore, LAN gaming centers may induce HFHL.

In contrast, subjects who attended concerts had a lower prevalence of HFHL than those who did not. Of 257 subjects who attended concerts in the past year, 154 (65.3%) went to a single concert, in marked contrast to the number of visits to LAN gaming centers. Occasional exposure to concert noise is less likely to affect hearing than repetitive noise. Also, the concert noise level would vary by the featured music genre. Last, those who attended concerts had higher family incomes than others; the negative association between concert attendance and HFHL is explained by the difference in income level.

Both HFHL and SFHL were negatively related to household income, consistent with the findings of previous studies. The National Health and Nutrition Examination Survey of 2005–2006 reported that school-aged children living in poverty had a higher prevalence of HL than other groups [11]. A Korean population-based study also reported an association between low family income and adolescent HL [13]. How household income affects hearing remains controversial. Some authors have suggested that any direct relationship between socioeconomic status and HL remains unproven, rather being attributable to other factors such as prematurity, low birth weight, or poor familial income attributable to parental hearing impairment [22, 23]. Other authors have found that adolescents of high socioeconomic status were more concerned about noisy activities and used more hearing-protection devices [24]. In our data, there was no association between household income and PLD use or volume. However, household income was negatively associated with LAN gaming center usage and was positively associated with Karaoke room usage and attending a concert. Comparing to the lowest income group, the highest income group had OR for LAN gaming center usage of 0.55 (95% CI: 0.30–0.99), OR for Karaoke room usage of 1.85 (95% CI: 1.004–3.41), and OR for attending a concert of 4.21 (95% CI: 2.28–7.79). The different pattern of LNE according to the household income may affect the different prevalence of HL.

We found that even slight HL among school-aged children is significantly associated with self-rated academic performance after adjusting for gender, grade, and household income. Previous studies found that educational performance was (negatively) associated with mild bilateral or unilateral HL [25–28]. Bess et al. reported that the grade retention rate (repeating one or more grades) of children with minimal sensorineural HL was significantly higher than the school-district norms, and that the educational performance of such children was significantly poorer than that of other children [29]. A recent, prospective cohort study showed that children with unilateral HL could catch up in terms of language and verbal intelligence skills over time but not in terms of academic performance [30]. School-aged children with minimal sensorineural HL exhibit poorer speech-recognition ability than those without HL [31]. Although some authors found no differences in terms of the effort devoted to hearing and the associated fatigue between children with minimal HL and others [32], the former children found listening demanding and experienced more listening-related fatigue, as did children with profound HL [33]. Furthermore, a population-based study in Australia showed that children with mild HL exhibited worse phonological short-term memory and phonological discrimination than others [34]. Therefore, differences in speech-recognition ability, short-term memory and listening-related fatigue may explain why children with slight HL exhibit poorer academic performance.

Hearing screening tests have been conducted in preschool and/or school-aged children in many countries [35, 36]. Pure tone audiometry at multiple frequencies, including 1, 2, and 4 kHz, is the usual method to screen school-age children, and the criteria for pass, refer, re-screen vary from 20 to 35 dB [36]. Currently, South Korea conducts simple hearing screening tests annually to all elementary school students in grades 1 and 4, and middle and high school students in grade 1. However, the screening test only records the presence or absence of a reaction in each ear when 40 dB of sound is delivered at a single frequency of 1 kHz. Therefore, it is difficult to detect a mild HL < 40 dB and/or HL limited to a specific frequency such as the ski-slope type of HL. The latest school hearing test report stated that the prevalence of “ear disease”, including HL, otitis media, and otitis externa is only 0.2% [37]. Thus, the current hearing test does not detect the slight HL affecting > 15% of all adolescents. Consequently, neither risk-factor control nor relevant education are yet possible. Given the fact that even slight HL can affect academic performance, and thus the self-esteem, of adolescents, we strongly recommend that careful hearing tests at several frequencies should be offered to middle and high school students [38].

Our study had certain limitations. Some questions required long-term memory recall (perinatal history of the child and residence history) and students often do not know their exact medical history. Thus, more complicated questions were answered by the parents, while students were asked about their major medical history in the past year. In addition, causal inferences cannot be drawn due to the cross-sectional nature of the study design. The school participation rate was low because of the complexity of the methods, which required active involvement of students, parents, and teachers. The audiological tests were conducted at the mobile soundproof booth in schools for convenience; this may have caused an increased prevalence of slight HL at the speech frequencies, due to uncontrolled environmental vibrations and excessive noises from the nearby playground or school buildings.

Despite these drawbacks, this study is noteworthy in that it assessed, through detailed audiometry, the actual prevalence of HL and HFHL in adolescents who were regarded as normal by conventional hearing screening testing. In addition, the authors analyzed the association between various types of LNE and HL. As LNE in adolescents is increasing, and is expected to increase further in the years to come, the results of our study constitute useful information for preventing HL in adolescents.

## Conclusions

About 17% (one-sixth) of Korean adolescents exhibit at least slight HL. Frequent exposure to loud, high-intensity leisure noise can affect the hearing threshold. HL may be associated with poor school performance. It is important to avoid excessive exposure to leisure noise when seeking to prevent HL in adolescents.

## Supporting information

S1 Text Questionnaire for students. (PDF)

S2 Text Questionnaire for parents. (PDF)

S1 Table Data set. (XLSX)
