# Supplementary material for: Hearing loss in Korean adolescents: The prevalence thereof and its association with leisure noise exposure
Source: PLoS One. 2019 Jan 4;14(1):e0209254. doi: 10.1371/journal.pone.0209254 (PMC6319738; doi:10.1371/journal.pone.0209254)
Supplement: S1 Text — (PDF) [file pone.0209254.s001.pdf]

This survey gathers health information before your check-up and will be used as a reference during consultation. Confidentiality is guaranteed. Please be sure to answer all questions candidly and sincerely to help protect and improve your health. Please fill in the questions by yourself. If you need help, consult a parent or guardian.

1. This is a question about ENT disease.

Disease	Has been diagnosed by a doctor (lifetime)	Treatment during the last 2 weeks (surgery, medication, etc.)
Rhinosinusitis	□ Yes □ No	□ Yes □ No
Ear infection	□ Yes □ No	□ Yes □ No

2. Choose the sentence that best describes your hearing.

Item	Select
No problem ⇨ Go to question no. 4.	□
Somewhat uncomfortable	□
Very uncomfortable	□
I can’t hear at all.	□

3. Are you currently using a hearing aid or cochlear implant?

Item	Select
Yes	□
I have one but rarely use it	□
No	□

4. Have you ever heard a sound in your ear in the past year? (a low buzzing, buzzing, humming, or machine-like sound, etc.)

Item	Select
Yes	□
No ⇨ Go to question no. 6	□
I don’t remember ⇨ Go to question no. 6	□

5. How uncomfortable does the sound in your ear make you?

Item	Select
No problem	□
I am annoyed and bothered	□
I find it hard to sleep.	□

6. The following questions are about your use of earphones and headphones. Please answer the questions.

Items	Answers
Do you use any portable music or video playback device such as a smartphone, MP3 player, a PMP, etc?	□Yes<br>□No ⇨ Go to question no. 7
When did you start using it?	______Years of age
What device do you usually use?	□Earphones □Headphones
Has anyone nearby ever asked you to turn down the volume when you were using a portable playback device?	□Yes □No

What volume level do you set when using a portable music player?

0 1 2 3 4 5 6 7 8 9 10

Questionnaire

(for middle/high school students)

7. Please describe your experience with the following equipment or facilities during the past year.

Item	Experienced or not	Age at first use	Frequency of use	Average time spent on each visit
LAN gaming center	□	____ years old	_____ times/month	___hours
Karaoke	□	____ years old	_____ times/month	___hours
Concert	□	____ years old	_____ times/month	___hours
Club, discotheque	□	____ years old	_____ times/month	___hours

8. Please fill out the location of your seat in class for the past 3 years, and explain why you are in that seat.

	Based on			Distance from lecture podium
	Height	Grade	Choice	
1 year ago	□	□	□	Very close Close Middle Far Very far
2 years ago	□	□	□	Very close Close Middle Far Very far
3 years ago	□	□	□	Very close Close Middle Far Very far

9. Please rate your grades for the past three years.

	Grade
1 year ago	Highest High Middle Low Lowest
2 years ago	Highest High Middle Low Lowest
3 years ago	Highest High Middle Low Lowest

10. The following questions explore your smoking-related behavior.

1) Have you ever smoked a cigarette, including just one or two puffs?
□ Never so far ⇨ Go to question no. 11
□ Yes
2) When was the first time you smoked a cigarette, including just one or two puffs?
_________years of age
3) On how many days did you smoke cigarettes during the last month?
_________days
□ Have not smoked during the last month ⇨ Go to question no. 11
4) How many cigarettes did you smoke daily (on average) during the last month?
Average __________cigarettes

Student’s personal information	School name	school	
	Grade/Class	grade class	
	Recipient Code		
	Gender	Male	Female

11. The following questions explore your drinking-related behavior.

1) Have you ever drunk more than one drink?
※ Excepting a sip or two during memorial services

□ Have never drunk ⇨ Go to question no.13
□ Yes

2) When was the first time you drank a whole cup of alcohol?
※ Excepting a sip or two during memorial services
__________ years of age

12. The following questions explore your drinking experience during the past year.

1) How often do you drink alcohol?

□ Did not drink at all for the past year ⇨ Go to question no. 13
□ Less than once a month
□ About once a month
□ 2–4 times a month
□ 2–3 times a week
□ More than 4 times a week

2) How many drinks do you consume at one time?
※ Each drink of liquor is counted regardless of alcohol type. One can of beer (355 mL) is equivalent to 1.6 cups of beer.

□ 1–2 cups
□ 3–4 cups
□ 5–6 cups
□ 7–9 cups
□ More than 10 cups

13. What is your current height (cm) and weight (kg)?

1. Height (cm)	2. Weight (kg)

This survey gathers health information before your check-up and will be used as a reference during consultation. Confidentiality is guaranteed. Please be sure to answer all questions candidly and sincerely to help protect and improve your health. Please fill in the questions by yourself. If you need help, consult a parent or guardian.

1. If any of your family members have ever been treated or diagnosed with the following diseases, please mark "V".

Family medical history	Yes	Who	Name of disease
Hypertension or cerebral stroke (stroke)			
Heart diseases such as angina, myocardial infarction, and/or heart failure			
Diabetes			
Cancer			
Liver disease			
Tuberculosis			
A psychiatric disorder			

2. If you have had any of the following diseases during the past year, please mark "V" and write down the specific disease name.

Medical history		Yes	Name of disease
Digestive system	Gastritis or peptic ulcer		
	Constipation		
	Enteritis		
Respiratory system	Asthma		
	Sinusitis		
	Allergic rhinitis or chronic rhinitis		
	Tonsillar hypertrophy		
Eye / Ear	Eye disease, strabismus, etc.		
	Ear infection		
	Hearing impairment		
	Other ear diseases (tinnitus, etc.)		
Skin	Atopic dermatosis		
	Other skin diseases		
Circulatory system	Anemia		
	Hypertension		
Musculo-skeletal system	Scoliosis		
	Disk or back pain		
	Disorder of limb movement		
	Injury or accident		
Other diseases	Pediatric psychiatric disorder		
	Tuberculosis		
	Drug allergy		
	Diabetes		
	Headache (except migraine)		
	Convulsion or seizures (spasms)		
	Speech disorder (including stuttering)		
	Hepatitis (from childhood)		
	Surgical experience (from childhood)		
	Hospitalization experience (from childhood)		
	Other diseases (addiction, suicide attempt, etc.)		

Questionnaire

(for middle/high school students)

3. Please mark "V" for all the symptoms you have experienced in the past month.

Item	Symptoms	Yes	No
Condition	Catch colds easily.		
	I lack strength and I get tired easily.		
	I think I am not healthy.		
Respiratory	My nose and eyes are itchy, I sneeze, and sometimes I have a clear runny nose.		
	I experience shortness of breath and I hear whistles from my chest.		
	I have yellow sputum when I cough.		
	I usually have trouble breathing through my nose because my nose is blocked.		
	I am told that I often snore badly.		
	I often have fever and a sore throat.		
	There is a lump in my neck.		
Circulatory	I get out of breath, even with little exercise, compared with others.		
	My complexion is bad and my heart pounds even when I am at rest.		
Digestive system	I have a burning feeling or pain in my stomach.		
	I feel stuffy or full.		
	My lower belly is painful or I often have diarrhea.		
	My stomach feels tight or bloated.		
Mental health	I feel hopeless because I am sad or depressed.		
	I often do not want to go to school.		
	I have seriously considered suicide or attempted suicide.		
	I often feel nervous as if I am going crazy.		
	I am not calm. I am too active, which can interfere with the activities of other children.		
Blood	I often have nosebleeds and the bleeding does not stop quickly if I am injured.		
	I get bruised easily.		
Other symptoms	My headache or migraine is severe.		
	My ears are sore or I have an ear discharge.		
	I hear buzzing in my ear.		
	My jaw joints are sore or I cannot open my mouth well.		
	I have a throbbing pain in my neck/waist/knee etc.		
	(For girls) My menstrual cramps are severe.		

Anything you want to say to your doctor?	

Student's personal information	School name		school
	Grade/ Class	grade	class
	Recipient Code		
	Gender	Male	Female

4. The following questions are about your health-related behavior. Please use "V" to answer.

Healthy lifestyle		Yes	No
Diet	I eat breakfast regularly.		
	I drink milk or dairy products every day.		
	I eat vegetables and fruits every day.		
	I rarely eat sweet or salty food.		
	I consume soft drinks, hamburgers, pizza, or snacks almost every day.		
	I skip meals or take drugs to lose weight.		
Personal hygiene	I wash my hands with soap before eating or after going out.		
	I brush my teeth more than twice a day.		
Exercise	I exercise intensely at least three times a week.		
	I allocate study and exercise time well. I feel refreshed after sleeping.		
Safety	I wear a seat belt when I ride in a car.		
	I wear a helmet or protective gear when I use inline skates, roller blades, skateboards, bicycles, etc.		
Internet	I use the internet or play games more than 2 hours a day.		
	I often watch pornography or chat on adult sites.		
Home & school life	I have been bullied or isolated by my friends over the past year.		
	I have a person to talk to when I have worries or problems.		
	I am worried about problems in my family.		
	I have seriously considered running away from home during the past year.		
	My safety is threatened by violence at home or school.		
	I have carried a knife/club/nunchaku as a weapon.		
Drug use	I have smoked during the past month.		
	I have drunk alcohol in the past month.		
	I have used marijuana or psychotropic drugs to hallucinate.		
Sexuality	I worry about gender issues.		
	I have been abused physically, mentally, and/or sexually.		
※ I'd like to receive consultation on my worries and troublesome matters.			
(At school's discretion)			

이 설문조사는 건강검사에 앞서 여러분의 건강상태를 미리 알아보고 진찰을 받을 때 참고하기 위한 것입니다. 비밀이 보장되므로 설문내용에 솔직하고 성실하게 답변해서 여러분 자신의 건강보호 및 향상에 도움이 되도록 합시다. 본인이 작성하되 잘 모르는 문항은 부모님(보호자)과 상의하여 기재하기 바랍니다.

1. 이비인후질환에 대한 질문입니다.

질병	의사에게 진단을 받았음 (평생)	최근 2주간 치료여부 (수술, 약물 등)
비부비동염(축농증)	☐ 예 ☐ 아니오	☐ 예 ☐ 아니오
중이염	☐ 예 ☐ 아니오	☐ 예 ☐ 아니오

2. 다음 중 본인의 청력을 가장 잘 표현한 문장을 선택하십시오.

문항	선택
불편하지 않다 ⇨ 4번 문항으로 넘어가십시오	☐
약간 불편하다	☐
많이 불편하다	☐
전혀 들리지 않는다	☐

3. 현재 보청기나 인공와우를 사용하고 계십니까?

문항	선택
예	☐
있지만 거의 사용 안 한다	☐
아니오	☐

4. 최근 1년 이내, 귀에서 소리가 난 적이 있습니까? (지지지, 삐, 웅웅, 기계소리 등)

문항	선택
예	☐
아니오 ⇨ 6번 문항으로 넘어가십시오	☐
기억이 나지 않는다 ⇨ 6번 문항으로 넘어가십시오	☐

5. 귀에서 나는 소리가 생활에 얼마나 불편하신지요?

문항	선택
불편하지 않다	☐
성가시고 신경이 쓰인다	☐
잠을 이루기가 힘들 정도이다	☐

6. 다음은 이어폰·헤드폰 이용에 관한 문항입니다. 질문에 응답하여 주십시오.

문항	Answers
스마트폰, MP3 플레이어, PMP 등의 이동식 음악·동영상 재생장치를 이용하십니까?	☐예<br>☐아니오⇨7번 문항으로 넘어가십시오
언제부터 이용하셨습니까?	만______세
주로 어떤 장비를 이용하십니까?	☐이어폰 ☐헤드폰
이동식 재생장치를 이용할 때 주변 사람에게 소리를 줄여달라는 이야기를 들은 적이 있습니까?	☐예 ☐아니오

이동식 음악재생장치를 이용할 때 어느 정도의 음량으로 이용하십니까?

0 1 2 3 4 5 6 7 8 9 10

문 진 표

(중·고등학생용)

7. 최근 1년 동안의 다음의 기기 또는 시설을 이용한 경험에 대하여 기입하여 주십시오.

항목	이용여부	최초 이용 시기	이용 빈도	1회 평균 시간
PC 방	☐	만___세	월_____회	___시간
노래방	☐	만___세	월_____회	___시간
콘서트장	☐	만___세	연간_____회	___시간
클럽, 디스코텍	☐	만___세	연간_____회	___시간

8. 지난 3년간 학생의 좌석의 위치와 좌석의 기준을 기입하여 주십시오.

	기준			교탁과의 거리
	키	성적	자율	
1년 전	☐	☐	☐	매우 가깝다 가깝다 중간쯤이다 멀다 매우 멀다
2년 전	☐	☐	☐	매우 가깝다 가깝다 중간쯤이다 멀다 매우 멀다
3년 전	☐	☐	☐	매우 가깝다 가깝다 중간쯤이다 멀다 매우 멀다

9. 지난 3년간 학생의 학급 내 성적을 체크하여 주십시오.

	성적
1년 전	최상위권 상위권 중위권 하위권 최하위권
2년 전	최상위권 상위권 중위권 하위권 최하위권
3년 전	최상위권 상위권 중위권 하위권 최하위권

10. 다음은 여러분의 흡연과 관련된 행동에 대한 질문입니다.

1) 지금까지 담배를 한 두 모금이라도 피워 본 적이 있습니까?
☐ 지금까지 없음 ⇨ 11번 문항으로 넘어가십시오
☐ 있음

2) 처음으로 담배를 한 두 모금이라도 피워 본 때는 언제입니까?
만_________세

3) 최근 1달 동안 담배를 한 대 라도 피운 날은 며칠입니까?
_________일
☐ 최근 1달 동안 담배를 피운 적이 없음⇨ 11번 문항으로 넘어가십시오

4) 최근 1달 동안 담배를 피운 날, 하루 평균 몇 개비나 피웠습니까?
평균__________개비

학생 인적 사항	학 교 명	학교	
	학년/ 반	학년 반	
	대상자 코드		
	성 별	남	여

11. 다음은 여러분의 음주와 관련된 행동에 대한 질문입니다.

1) 지금까지 살아오면서 1잔 이상의 술을 마신 적이 있습니까?
※ 단, 제사·차례 때 몇 모금 마셔본 것은 제외합니다

☐ 술을 마셔 본 적 없음 ⇨ 13번 문항으로 넘어가십시오
☐ 있음

2) 처음으로 술 1잔을 모두 마셔본 적은 언제입니까?
※ 단, 제사·차례 때 몇 모금 마셔본 것은 제외합니다
만___________ 세

12. 다음은 여러분의 최근 1년 동안의 음주(술) 경험에 대한 질문입니다.

1) 술을 얼마나 자주 마십니까?

☐ 최근 1년간 전혀 마시지 않았다 ⇨ 13번 문항으로 넘어가십시오
☐ 한달에 1번 미만
☐ 한달에 1번 정도
☐ 한달에 2~4번
☐ 일주일에 2~3번 정도
☐ 일주일에 4번 이상

2) 한번에 술을 얼마나 마십니까?
※ 소주, 양주 구분 없이 각각의 술잔으로 계산합니다.
단, 캔맥주 1개(355 cc)는 맥주 1.6잔과 같습니다.
☐ 1~2잔
☐ 3~4잔
☐ 5~6잔
☐ 7~9잔
☐ 10잔 이상

14. 현재 귀하의 키(신장cm)와 몸무게(체중kg)는 얼마입니까?

1. 키 (cm)	2. 몸무게 (kg)

이 설문조사는 건강검사에 앞서 여러분의 건강상태를 미리 알아보고 진찰을 받을 때 참고하기 위한 것입니다. 비밀이 보장되므로 설문내용에 솔직하고 성실하게 답변해서 여러분 자신의 건강보호 및 향상에 도움이 되도록 합시다. 본인이 작성하되 잘 모르는 문항은 부모님(보호자)과 상의하여 기재하기 바랍니다.

1. 가족 중 다음과 같은 질환을 치료받거나 진단받은 사람이 있으면 해당 질환에 "V" 표시를 하여 주십시오.

가족의 의학적 병력	있음	누가	질병명
고혈압이나 뇌졸증(중풍)			
협심증.심근경색.심부전 등 심장질환			
당뇨병			
암			
간질환			
결핵			
정신질환			

2. 최근 1년 동안 다음과 같은 질병을 앓은 적이 있으면 모두 "V" 표시를 하고 구체적인 질병명을 써 주십시오.

질병의 과거력		있음	질병명
소화기계	위염 또는 소화성궤양		
	변비		
	장염		
호흡기계	천식		
	축농증		
	알레르기성비염 또는 만성비염		
	편도선비대		
눈·귀	안질환.사시 등		
	중이염		
	청력장애		
	그 밖의 귀 질환(이명 등)		
피부	아토피피부병		
	그 밖의 피부질환		
순환기계	빈혈		
	고혈압		
근골격계	척추측만증		
	디스크나 요통		
	사지운동의 장애		
	외상이나 사고		
그 밖의 질환 등	소아정신과 질환		
	결핵		
	약물알레르기		
	당뇨병		
	두통(편두통 제외)		
	경련이나 발작(경기)		
	언어장애(말더듬 포함)		
	간염 (어려서부터)		
	수술한 경험(어려서부터)		
	입원한 경험(어려서부터)		
	그 밖의 질환(중독.자살기도 등)		

문 진 표

(중·고등학생용)

3. 최근 한 달간 경험한 증상에 모두 "V"표시를 하여 주십시오.

항목	나타나는 증상	예	아니오
전신 상태	감기에 잘 걸린다.		
	온 몸에 힘이 없고 쉽게 피로하다.		
	건강하지 않다고 생각한다.		
호흡기	재채기와 함께 코와 눈이 가렵고 맑은 콧물이 흐를 때가 있다.		
	숨쉴 때 숨이 가쁘면서 가슴에서 쌕쌕하는 소리나 휘파람 소리가 들릴 때가 많다.		
	기침과 함께 누런 가래가 올라온다.		
	평소 코로 숨쉬기가 불편하고 코가 막힌다.		
	코를 심하게 곤다는 말을 듣는다.		
	열이 많이 나면서 목이 따가울 때가 자주 있다.		
	목에서 몽우리가 만져진다.		
순환기	운동을 조금만 해도 다른 사람보다 숨이 심하게 차다.		
	혈색이 안 좋고 가만히 있어도 심장이 두근거린다.		
소화기	속이 쓰리거나 아플 때가 있다.		
	속이 답답하거나 가득 찬 듯한 느낌이 있다.		
	아랫배가 살살 아프거나 설사를 자주 한다.		
	배가 팽팽하거나 가스가 찬 것 같다.		
정신 건강	기분이 처지거나 우울해서 희망이 없다는 느낌이 든다.		
	학교를 가고 싶지 않을 때가 자주 있다		
	자살을 심각하게 생각하거나 자살을 시도한 적이 있다.		
	미칠 것 같은 불안을 자주 느낀다.		
	차분하지 않고 지나치게 활동적이라 다른 아이에게 방해가 되는 경우가 있다.		
혈액	코피가 자주 나고 다치면 피가 잘 멈추지 않는다.		
	몸에 멍이 잘 든다.		
그 밖의 증상	두통이나 편두통이 심하다.		
	귓속이 아프거나 귀에서 분비물이 나온다.		
	귀에서 매미우는 소리나 윙하는 소리가 들린다.		
	턱관절이 아프거나 입이 잘 벌어지지 않는다.		
	목.허리.무릎 등이 쑤시거나 아프다.		
	(여학생) 생리통이 심하다.		

특별히 의사선생님께 하고 싶은 말	

학생 인 적 사 항	학 교 명	학교	
	학년/ 반	학년 반	
	대상자 코드		
	성 별	남	여

4. 다음은 여러분의 건강생활과 관련된 행동에 대한 질문입니다. 자신에게 해당되는 질문에 "V"표시를 하여 주십시오.

건 강 생 활 행 동		예	아니오
식생활	아침은 규칙적으로 먹는 편이다.		
	매일 우유나 유제품을 먹는다.		
	매일 채소와 과일을 먹는다.		
	단음식이나 짠 음식을 거의 먹지 않는다.		
	청량음료.햄버거.피자 또는 과자를 거의 매일 먹는다.		
	체중을 줄이기 위해 굶거나 약을 먹는다.		
개인위생	식사하기 전이나 외출 후 돌아와서 비누로 손을 씻는다.		
	하루에 두 번 이상 이를 닦는다.		
운동	주 3회 이상 땀이 나거나 숨이 찰 정도로 운동을 하거나 일을 한다.		
	공부와 운동을 잘 안배하여 자고 나면 개운하다.		
안전	자동차를 탈 때 안전벨트를 맨다.		
	인라인스케이트, 롤러블레이드, 스케이트보드, 자전거 등을 탈 때 헬멧이나 보호구를 착용한다.		
인터넷	인터넷이나 게임을 하루에 2시간 이상 한다.		
	음란물을 보거나 성인사이트에서 채팅을 자주 한다.		
가정 및 학교생활	지난 1년 동안 친구들에게 괴롭힘이나 따돌림을 당한 적이 있다.		
	고민이 있거나 괴로울 때 의논할 수 있는 사람이 있다.		
	가정(가족)내의 문제에 대해 걱정이 된다.		
	지난 1년 동안 가출하는 것을 심각하게 생각해 본 적이 있다.		
	가정이나 학교에서 폭력으로 인해 자신의 안전이 위협을 받고 있다.		
	무기로 사용할 목적으로 칼.몽둥이.쌍절곤 등을 가지고 다닌 적이 있다.		
약물	지난 1개월 동안 담배를 피운 적이 있다.		
	지난 1개월 동안 술을 마신 적이 있다.		
	환각을 목적으로 불법으로 대마초나 향정신성 약물을 사용한 적이 있다.		
성	성문제에 대해서 고민이 된다.		
	육체적.정신적.성적으로 학대를 당한 적이 있다.		
※ 고민이나 괴로운 일에 대해 상담을 받고 싶다.			
(학교 재량란)			
