# Supplementary material for: Hearing loss in Korean adolescents: The prevalence thereof and its association with leisure noise exposure
Source: PLoS One. 2019 Jan 4;14(1):e0209254. doi: 10.1371/journal.pone.0209254 (PMC6319738; doi:10.1371/journal.pone.0209254)
Supplement: S2 Text — (PDF) [file pone.0209254.s002.pdf]

This questionnaire explores parental/guardian health conditions in advance of a health checkup. The answers will help students' parents to live a healthy life and improve their quality-of-life. Please take the time to respond.

1. The following questions are about your health condition and illnesses that you have suffered or for which you have been treated.

(1) Have you been diagnosed with (name of the disease) from a doctor?
(2) Treatment for (name of the disease)
① **Fully recovered; no treatment** or
② **Not fully recovered; but no treatment** or
③ **Currently being treated.**

Name of disease	Diagnosed by a doctor	Treatment: Select from ①②③ above
Cancer (area:)		
Hypertension		
Diabetes		
Hyperlipidemia		
Heart disease such as angina, myocardial infarction, or heart failure		
Osteoarthritis (degenerative arthritis)		
Asthma		
Chronic obstructive pulmonary disease		
Other ( )		

2. The following questions are about your health behavior.

1) Questions about smoking. Do you smoke?
☐① I have never smoked
☐② I smoked before but I quit (____years ago)
☐③ Currently smoke (Go to question **A**)
A) If you currently smoke
a. On average, how many cigarettes do you smoke per day? ________cigarettes
b. How many years have you smoked? Total______years

2) Questions about drinking habits. What is your drinking status?
☐① Never drink alcohol
☐② Quit (____years ago)
☐③ Currently drink alcohol (average number of days per week for the past month: _____days/week)

3) How many hours a day do you sleep on average per day?
_____hours

4) Do you think that your sleep hours are sufficient?
☐① Very sufficient ☐② Sufficient.
☐③ Not sufficient. ☐④ Very insufficient.

Questionnaire
(For parents)

4. The following question is about your cancer screening. Please use "V" to indicate when you underwent the last test for each item.

	Less than 1 year	1–2 years	2–5 years	More than 5 years	Never been tested
**Stomach cancer** examination (Gastroscopy or upper gastro-intestinal series)	☐	☐	☐	☐	☐
**Breast cancer** examination (Including mammography or breast ultrasound)	☐	☐	☐	☐	☐
**Cervical cancer** examination (Pap smear)	☐	☐	☐	☐	☐
**Colon cancer** examination (Colonoscopy or double-contrast barium examination and S-colonoscopy)	☐	☐	☐	☐	☐

5. Please mark "V" the last time you underwent each of the following tests.

	Less than 1 year	1–2 years	2–5 years	More than 5 years	Never been tested
Blood pressure test	☐	☐	☐	☐	☐
Diabetes test	☐	☐	☐	☐	☐
Cholesterol test	☐	☐	☐	☐	☐
Urine test (proteinuria test)	☐	☐	☐	☐	☐
Fundoscopy (eye test)	☐	☐	☐	☐	☐

6. Have you undergone influenza (flu) vaccination during the past year?

Influenza (flu) vaccinations are usually performed in the fall and prevent influenza (flu).

☐① Yes ☐② No ☐③ I don't know

7. Today, what is your height (cm) and weight (kg)?

1. Height (cm)	2. Weight (kg)

Student's personal information	School name	school	
	Grade/ Class	grade class	
	Recipient Code		
	Gender	Male	Female
Guardian's signature		Sign	

8. The following questions are about your physical activity during the past week.

1) During the last week, how many days did you spend more than 10 minutes doing intense physical activity with a lot of breathing and a heart rate increase?_________days/week,
Active time for that day __________hours___minutes

2) During the last week, how many days did you spend more than 10 minutes doing physical activity with a slightly increased breathing and heart rate?__________days/week,
Active time for that day ___________hours___minutes

* Intense physical activity including occupational and physical activity, tennis doubles, volleyball, badminton, table tennis, swimming, yoga, and gymnastics (except walking)

3) How many days a week have you walked more than 10 minutes a day?_________days/week,
Average hours per day ________hours___minutes
* Including commuting, and walking to travel or to exercise.

9. Please use "V" to answer each item.

	Always	Sometimes	No
I can ask family for help if I have problems or difficulties.	☐	☐	☐
I am satisfied with how my family communicates and shares my difficulties to help solve problems.	☐	☐	☐
My family helps me when I try to do something new or make a suggestion.	☐	☐	☐
I am satisfied with how my family responds to my feelings, such as anger, sadness, and love.	☐	☐	☐
I think that the way I spend my leisure time with my family is good for my family.	☐	☐	☐

10. What is your religion?
☐①Christian ☐②Buddhist ☐③Catholic ☐④Other ☐⑤No religion

11. What is your family's average monthly income?
☐①Less than 1 million won ☐②100–200 million won
☐③200–300 million won ☐④300–400 million won
☐⑤Over 400 million won

This questionnaire explores parental/guardian health conditions in advance of a health checkup. The answers will be help student's parents to live a healthy life and improve the quality-of-life. Please take the time to respond.

1. The following items refer to the health of the child in terms of environmental problems such as fine dust in the residence.
Please write all your past addresses and the times spent there.

No.	Address	Period
Example	Seoul Special city/Metropolitan city/Province Jongno City/ Town/ District Yeongeon Eup/Myeon/Dong 28 Department of Preventive Medicine, Seoul National University College of Medicine	1998. 5 – 2003. 3
1	______Special city/Metropolitan city/Province ______City/ Town/ District ______Eup/Myeon/Dong________________	–
2	______Special city/Metropolitan city/Province ______City/ Town/ District ______Eup/Myeon/Dong________________	–
3	______Special city/Metropolitan city/Province ______City/ Town/ District ______Eup/Myeon/Dong________________	–
4	______Special city/Metropolitan city/Province ______City/ Town/ District ______Eup/Myeon/Dong________________	–
5	______Special city/Metropolitan city/Province ______City/ Town/ District ______Eup/Myeon/Dong________________	–
6	______Special city/Metropolitan city/Province ______City/ Town/ District ______Eup/Myeon/Dong________________	–
7	______Special city/Metropolitan city/Province ______City/ Town/ District ______Eup/Myeon/Dong________________	–
8	______Special city/Metropolitan city/Province ______City/ Town/ District ______Eup/Myeon/Dong________________	–

Questionnaire

(for parents)

2. The following questions are about your child's past health.

1) If any of the following items were applicable to your newborn child, please check and fill in the details.

- ☐ During pregnancy, was the mother infected with rubella, CMV, toxoplasmosis, etc.? (Details:____ ____ ________________)
- ☐ Birth weight less than 1.5 kg
- ☐ Plasma exchange because of newborn jaundice
- ☐ Apgar score below 4 at 5 minutes after birth
- ☐ Diagnosis of a doctor in terms of head, neck, or ear abnormalities (Details:____ ____ ________________)
- ☐ Admitted to neonatal intensive care unit or intensive care unit (Hospitalization period :_______months__________days)
- ☐ Home oxygen therapy
- ☐ None of the above

2) If your child has any of the following diseases, check the corresponding box.

	Diagnosed by a doctor	First time diagnosed (years old)	Ongoing	Currently receiving treatment
**Diabetes**	☐		☐	☐
**Hypertension**	☐		☐	☐
**Myocardial infarction**	☐		☐	☐
**Angina**	☐		☐	☐

3. If your child's family or relatives (parents, siblings, grandparents, uncles, cousins) suffer from hearing loss, please indicate this below.

Relationship	Current age (years)	Permanent hearing loss from birth	Permanent hearing loss since adolescence	Deaf before 60 years of age	Deaf after 60 years of age	Uses hearing aid or sign language
		☐	☐	☐	☐	☐
		☐	☐	☐	☐	☐
		☐	☐	☐	☐	☐
		☐	☐	☐	☐	☐
		☐	☐	☐	☐	☐

Student's personal information	School name	school	
	Grade/ Class	grade	class
	Recipient Code		
	Gender	Male	Female
Parent/guardian's signature		Sign	

이 설문조사는 건강검사에 앞서 학생 및 보호자(부모)의 건강상태를 미리 알아보고 진찰을 받을 때 참고하기 위한 것입니다. 특히, 보호자용 문진표의 경우, 학생의 보호자들이 건강한 삶을 영위하고, 삶의 질을 향상시키는데 기반이 되는 귀중한 자료로 활용될 수 있습니다. 잠시 시간을 내어 응답해 주시면 대단히 감사하겠습니다.

1. 다음은 귀하(보호자)의 건강 상태 및 앓고 계시거나 치료 받으신 질환에 대한 질문입니다.

(1) (질병명)은 의사로부터 진단을 받으신 것입니까?
(2) (질병명)을 위한 치료를
① 완치되어 치료받지 않고 있음 혹은
② 완치되지 않았지만 치료받지 않고 있음 혹은
③ 현재 치료 받고 있음 중에 골라 주십시오.

질병명	의사에게 진단받음	치료 여부 ①②③ 중 고름
암 (부위:)		
고혈압		
당뇨병		
고지혈증		
협심증.심근경색.심부전 등 심장질환		
골관절염 (퇴행성 관절염)		
천식		
만성폐쇄성폐질환		
기타 ( )		

2. 다음은 귀하(보호자)의 건강행태에 대한 질문입니다.

1) **흡연**에 관한 질문입니다. 담배를 피우십니까?
☐ ① 전혀 피운 적이 없다
☐ ② 이전에 피웠으나 끊었다 (금연 한 지 몇 ___년 전)
☐ ③ 현재도 피우고 있다 (**가** 문항으로)
가) 현재도 흡연을 하신다면
㉮ 하루 평균 몇 개비나 피웠습니까? ______개비
㉯ 몇 년째 담배를 피우시고 계십니까? 총______년

2) **음주(술)습관**에 관한 질문입니다. 귀하의 음주상태는 어떻습니까?
☐ ① 원래부터 음주하지 않음
☐ ② 끊었음(끊은 지 ______년 되었음)
☐ ③ 현재도 음주 중 (지난 한달 동안 1주간 평균 음주 횟수 ____일/주)

3) 하루 평균 몇 시간 정도 **수면**을 취하십니까? _____시간

4) 하루에 취하고 있는 수면이 피로회복에 충분하다고 생각하십니까?
☐ ① 매우 충분하다. ☐ ② 충분한 편이다.
☐ ③ 불충분한 편이다. ☐ ④ 대단히 불충분하다.

문 진 표

(부모용)

4. 다음은 귀하(보호자)의 암검진에 대한 질문입니다. 각각에 대해 가장 마지막으로 암검사를 받은 때에 "V"표시를 하여 주십시오.

	1년 미만	1년 이상 2년 미만	2년 이상 5년 미만	5년 이상	한 번도 검사 받은 바 없음
**위암** 검사 (위내시경 또는 조영술)	☐	☐	☐	☐	☐
**유방암** 검사 (유방촬영 또는유방초음파 포함)	☐	☐	☐	☐	☐
**자궁 경부암** 검사 (자궁질경부세포도말검사)	☐	☐	☐	☐	☐
**대장암** 검사 (대장내시경검사 또는 이중조영바륨검사와 에스결장경검사)	☐	☐	☐	☐	☐

5. 다음 각각의 검사를 귀하(보호자)께서 마지막으로 받은 때에 "V"표시를 하여 주십시오.

	1년 미만	1년 이상 2년 미만	2년 이상 5년 미만	5년 이상	한 번도 검사 받은 바 없음
혈압측정	☐	☐	☐	☐	☐
당뇨검사	☐	☐	☐	☐	☐
콜레스테롤 검사	☐	☐	☐	☐	☐
소변검사 (단백뇨 검사)	☐	☐	☐	☐	☐
안저검사 (눈 검사)	☐	☐	☐	☐	☐

6. 지난 1년 동안, 인플루엔자(독감)백신 혹은 인플루엔자(독감)예방접종을 받으신 적이 있습니까?

인플루엔자(독감)예방접종은 대개 가을에 이루어지며 인플루엔자(독감) 예방기에 인플루엔자(독감)을 예방합니다

☐① 예 ☐② 아니오 ☐③ 모르겠음

7. 오늘, 귀하의 키(신장cm)와 몸무게(체중kg)는 얼마입니까?

1. 키 (cm)	2. 몸무게 (kg)

학생 인 적 사 항	학 교 명	학교	
	학년/ 반	학년 반	
	대상자 코드		
	성 별	남	여

보호자 서명	서 명

8. 다음은 지난 일주일동안 귀하의 신체활동에 대한 질문입니다.

1) 지난 일주일 동안 평소보다 숨이 많이 가쁘고 심장박동이 많이 증가하는 격렬한 신체활동을
10분 이상 하신 날을 며칠입니까? ___________일/주 ,
그런 날 활동시간 ________시간 ___분

2) 지난 일주일 동안 평소보다 숨, 심장박동이 조금 증가하는 중증도 신체활동을 10분 이상하신 날은
며칠입니까? ____________일/주 ,
그런 날 활동시간 _________시간 ___분
* 직업활동 및 체육활동을 포함한 중증도 신체활동 : 복식테니스, 배구, 배드민턴, 탁구, 수영, 요가, 미용체조 등(걷기 제외)

3) 지난 일주일 동안 하루에 10분 이상 걸은 날은
며칠입니까? ____________일/주 ,
하루 평균 _________시간 ___분
* 출퇴근 시간 포함, 이동 및 운동을 위해 걷는 것 모두 포함

9. 다음의 각 질문에 귀하(보호자)의 경우에 해당되는 곳에 "V"표시를 하여 주십시오.

	항상 그렇다	때때로 그렇다	아니다
나는 나를 괴롭히거나 어려운 일이 생기면 가족에게 도움을 청할 수 있다.	☐	☐	☐
나는 가족이 나와 함께 문제 해결을 위한 대화나 어려움을 나누어 갖는 방법에 대하여 만족한다.	☐	☐	☐
나의 가족은 내가 새로운 일을 하려고 하거나 새로운 제안을 할 때 이를 따라주고 도와준다.	☐	☐	☐
나는 분노, 슬픔, 사랑 등 나의 감정에 대응하는 나의 가족의 반응에 만족한다.	☐	☐	☐
나는 가족과 함께 여가를 보내는 방법 중 현재 우리 가족이 취하는 방법들이 좋다고 생각한다.	☐	☐	☐

10. 귀하의 종교는 어떻게 되십니까?
☐ ① 기독교 ☐ ② 불교 ☐ ③ 천주교 ☐ ④ 기타 ☐ ⑤ 무교

11. 귀하 가족의 월평균 수입은 어떻게 되십니까?
☐ ① 100만원 미만 ☐ ②100-200만원 ☐ ③ 200-300만원
☐ ④ 300-400만원 ☐ ⑤ 400 만원 이상

이 설문조사는 건강검사에 앞서 학생 및 보호자(부모)의 건강상태를 미리 알아보고 청력 문제를 진찰하는데 참조하고 학생 청력 건강 개선의 기반이 되는 귀중한 자료로 활용될 수 있습니다. 잠시 시간을 내어 응답해 주시면 대단히 감사하겠습니다.

1. 다음은 거주지의 미세먼지 등의 환경문제와 자녀의 건강의 관련을 보기 위한 내용입니다. 다음 칸에 지난 모든 거주지의 주소 및 거주기간을 기입하여 주십시오.

순번	거주지 주소	거주기간
예	서울 (특별시) / 광역시 / 도 종로 시 / 군 (구)<br>연건 읍 / 면 (동) 28번지<br>*서울대학교 의과대학 예방의학교실*	1998. 5 ~ 2003. 3
1	_____특별시 / 광역시 / 도______시 / 군 구<br>_____읍 / 면 / 동_________	~
2	_____특별시 / 광역시 / 도______시 / 군 구<br>_____읍 / 면 / 동	~
3	_____특별시 / 광역시 / 도______시 / 군 구<br>_____읍 / 면 / 동	~
4	_____특별시 / 광역시 / 도______시 / 군 구<br>_____읍 / 면 / 동	~
5	_____특별시 / 광역시 / 도______시 / 군 구<br>_____읍 / 면 / 동	~
6	_____특별시 / 광역시 / 도______시 / 군 구<br>_____읍 / 면 / 동	~
7	_____특별시 / 광역시 / 도______시 / 군 구<br>_____읍 / 면 / 동	~
8	_____특별시 / 광역시 / 도______시 / 군 구<br>_____읍 / 면 / 동	~

문 진 표

(부모용)

2. 다음은 자녀분의 과거 건강에 대한 질문입니다.

1) 다음 항목 중 자녀가 신생아시기에 해당되는 경우가 있다면 체크하고 내용을 기입하여 주십시오.

☐	아이가 뱃속에 있을 때 풍진, 거대세포바이러스(CMV), 톡소플라즈마 등에 감염 (내용 : ______________________________)
☐	출생 체중이 1.5kg 이하
☐	신생아황달로 인해 혈장교환술 시행
☐	출생 후 5분 아프가점수가 4점 이하
☐	머리, 목, 귀의 이상이 있다는 의사의 진단<br>(내용: ______________________________)
☐	신생아중환자실이나 집중치료실에 입원<br>(입원기간 :______개월_________일)
☐	재택 산소치료
☐	위 항목 모두 해당 없음

2) 다음 중, 자녀가 현재까지 치료받은 질환이 있다면 해당되는 란에 체크하여 주십시오.

	의사에게 진단을 받았음	처음 진단 받은 시기	현재 앓고 있음	현재 치료 여부
당뇨병	☐		☐	☐
고혈압	☐		☐	☐
심근경색증	☐		☐	☐
협심증	☐		☐	☐

3. 자녀분의 가족 및 친척(부모님, 형제/자매, 조부/조모, 삼촌, 사촌분들) 중 청력저하가 있으신 분이 있으시다면, 아래의 칸에 기입하여 주십시오.

관계	현 나이 (만)	태어날 때부터 영구적인 청력저하	청소년기부터 영구적인 청력저하	60세 이전 귀가 멈	60세 이후 귀가 멈	보청기 또는 수화 사용
	만____세	☐	☐	☐	☐	☐
	만____세	☐	☐	☐	☐	☐
	만____세	☐	☐	☐	☐	☐
	만____세	☐	☐	☐	☐	☐
	만____세	☐	☐	☐	☐	☐

학생 인적 사항			
	학 교 명	학교	
	학년/ 반	학년 반	
	대상자 코드		
	성 별	남	여
보호자 서명		서 명	
